# Discovery of New Candidate Genes Related to Brain Development Using Protein Interaction Information

**DOI:** 10.1371/journal.pone.0118003

**Published:** 2015-01-30

**Authors:** Lei Chen, Chen Chu, Xiangyin Kong, Tao Huang, Yu-Dong Cai

**Affiliations:** 1 Institute of Systems Biology, Shanghai University, Shanghai 200444, People’s Republic of China; 2 College of Information Engineering, Shanghai Maritime University, Shanghai 201306, People’s Republic of China; 3 Institute of Biochemistry and Cell Biology, Shanghai Institutes for Biological Sciences, Chinese Academy of Sciences, Shanghai 200031, People’s Republic of China; 4 Institute of Health Sciences, Shanghai Institutes for Biological Sciences, Chinese Academy of Sciences, Shanghai 200025, People’s Republic of China; Huazhong University of Science and Technology, CHINA

## Abstract

Human brain development is a dramatic process composed of a series of complex and fine-tuned spatiotemporal gene expressions. A good comprehension of this process can assist us in developing the potential of our brain. However, we have only limited knowledge about the genes and gene functions that are involved in this biological process. Therefore, a substantial demand remains to discover new brain development-related genes and identify their biological functions. In this study, we aimed to discover new brain-development related genes by building a computational method. We referred to a series of computational methods used to discover new disease-related genes and developed a similar method. In this method, the shortest path algorithm was executed on a weighted graph that was constructed using protein-protein interactions. New candidate genes fell on at least one of the shortest paths connecting two known genes that are related to brain development. A randomization test was then adopted to filter positive discoveries. Of the final identified genes, several have been reported to be associated with brain development, indicating the effectiveness of the method, whereas several of the others may have potential roles in brain development.

## Introduction

Beginning with the segregation of neural and glial cells from other types of tissues, brain development is a dramatic process composed of a series of complex and fine-tuned spatiotemporal gene expressions. These gene expressions contribute to brain transformation that involves both microscopic and macroscopic changes [1,2]. Although all mammalian embryos develop in a fundamentally similar way, human brains show more sophisticated neural structures and circuits than other species, which gives rise to human intelligence. Therefore, understanding the process and mechanism of brain development is important. To explore this mystery, molecular and cellular biology approaches have widely been applied over the past decades and greatly advanced our understanding in this field. Many genes and pathways have been identified that play essential roles in the development of human brains. For example, the Wnt-Frizzled signaling pathway was shown to be the key element in physiological neural-stem cell proliferation and differentiation. Additionally, this pathway could also lead to the pathological generation of brain cancers [1,3]. Sox family transcription factors also provided important clues about the differentiation of neural stem cells into neurons and glia [2].

Despite these advances, the intricate brain development process remains largely unveiled, and a great demand is present to discover new genes and identify their functions. However, the discovery of new genes related to brain development only by biochemical experiments is challenging because we have to investigate each gene, resulting in a high cost and substantial time. Fortunately, with the existing knowledge of genes and pathways that relate to brain development, we can unearth common features among the genes and develop computational methods to screen for candidate genes that have stronger associations with brain development. This screening reduces the search space and provides new clues for future research. Various biological problems have been completely or partly settled by designing effective computational methods [4,5,6,7,8,9,10,11,12]. Encouraged by the success of these studies, the design of effective computational methods is a useful process to discover new candidate genes related to brain development.

Recently, a group of computational methods was proposed to discover new disease genes according to the current known disease genes. These methods have been applied to find new genes related to various diseases including lung cancer [13] and hepatocellular carcinoma [14]. We proposed a similar computational method to discover new candidate genes related to brain development. Additionally, several newly found genes have high probabilities of being novel genes related to brain development because they have been shown to be expressed in the brain and involved in several brain diseases. The findings in this study will introduce new directions for the investigation of the biological process of brain development.

## Materials and Methods

### Materials

The 94 human genes related to brain development, with experimental evidence from Gene Ontology (GO:0007420), were retrieved from http://amigo.geneontology.org/amigo/term/GO:0007420 (Accessed 2014 May 10) [15]. These genes were used to discover new candidate genes for brain development and are available in S1 File.

In addition, we also downloaded the 610 human genes that are related to brain development with evidence from Gene Ontology (GO:0007420) at the aforementioned website. These genes included the genes mentioned in the above paragraph. The remaining 516 genes were shown to have associations with brain development by other methods instead of direct experiments. The 516 genes were termed as inferred genes for brain development and are listed in S2 File. These genes will be used to compare with genes discovered in our method.

### Construction of the weighted graph based on protein-protein interactions

Protein-protein interactions provide useful information to investigate protein-related problems [16,17,18,19,20] because the interaction can reflect the association between proteins from nearly all cellular functions such as regulation of signaling and metabolic pathways, protein synthesis, DNA replication, gene translation, and immunological recognition [21]. In this study, we employed protein-protein interactions retrieved from STRING (Search Tool for the Retrieval of Interacting Genes/Proteins, http://string.embl.de/) [22], which have been successfully applied to previously construct several computational methods [16,20,23]. The obtained protein-protein interactions were derived from genomic contexts, high-throughput experiments, (conserved) coexpression and previous knowledge. Thus, they contain the direct and indirect associations between proteins. For each obtained protein-protein interaction, the entry contains two proteins and one score, in which the score can reflect the strength of the interaction from many aspects of proteins. For later formulation, the score of the interaction containing proteins *p*_1_ and *p*_2_ was denoted by *S*(*p*_1_, *p*_2_).

According to the protein-protein interactions retrieved from STRING, we constructed a weighted graph *G* = (*V*, *E*) as follows: *V* represented all human proteins occurring in protein-protein interactions retrieved from STRING, and *E* contained all pairs of nodes such that the corresponding proteins comprised a protein-protein interaction in STRING. Because the range of interaction scores is between 150 and 999, a weight was assigned to each edge, *e*, in *G* by 1000-*S*(*p*_1_, *p*_2_), where *p*_1_ and *p*_2_ were two corresponding proteins of the endpoints of edge *e*. The constructed graph *G* consisted of 18,600 nodes and 1,640,707 edges.

### Methods for discovery of new candidate genes

The discovery method was executed on the weighted graph *G* constructed in Section “Construction of the weighted graph based on protein-protein interactions”, which consisted of two stages: (I) execute the shortest path algorithm on *G* for finding new candidate genes and (II) construct a randomization test to filter these candidate genes. Readers can refer to previous studies [13,14] for the detailed procedures of the method and its principle. Here, we only provide a brief description.

**Stage I**. The well-known shortest path algorithm, Dijkstra’s algorithm [24], was executed on *G* to find all of the shortest paths which connect any two genes related to brain development, *i.e*., genes listed in S1 File. Certain gene occurring in at least one of these paths as an inner node was selected as a candidate gene for brain development. For the later randomization test, we also counted the number of paths containing each candidate gene as an inner node. This value was termed as betweenness in this study.

**Stage II**. The randomization test was executed by constructing 500 gene sets whose sizes were equal to that of the set consisting of human genes related to brain development. For each gene set, we executed the procedure in **Stage I** and counted the betweenness for each candidate gene. Another value, named the permutation FDR (False Discovery Rate), was then computed for each candidate gene, which was defined as “the number of gene sets on which the betweenness exceeded that on the gene set related to brain development”/500. Finally, candidate genes with permutation FDRs smaller than 0.05 were selected as significant candidate genes for brain development.

## Results and Discussion

**Fig 1:**
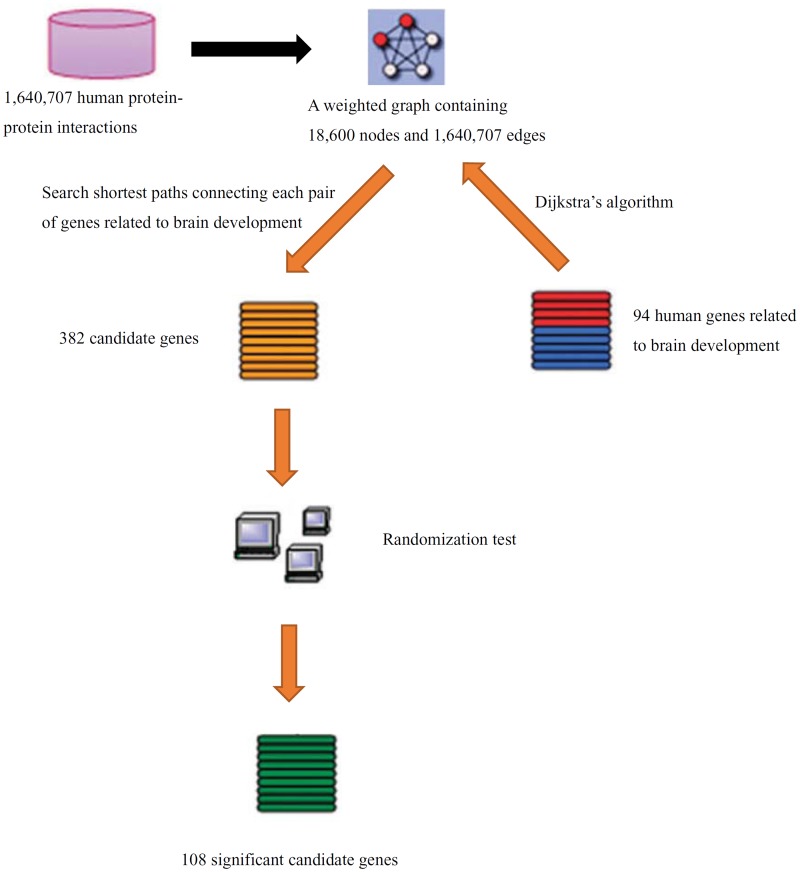
The workflow of the procedures for discovery of candidate genes related to brain development.

According to the procedures (including the construction of the weighted graph) described in Section “Construction of the weighted graph based on protein-protein interactions” and “Methods for discovery of new candidate genes”, some candidate genes for brain development can be identified. The workflow of these procedures is illustrated in Fig. 1.

### Candidate genes related to brain development

For the 94 genes listed in S1 File, the shortest paths that connect any two of the genes were obtained by applying the Dijkstra’s algorithm on *G*. Accordingly, we retrieved 382 candidate genes, which are listed in S1 Table. The betweenness of each candidate gene is also listed.

### Significant candidate genes related to brain development

According to the method, the 382 candidate genes were filtered by a randomization test, and the permutation FDR of each candidate gene was calculated, the results of which are available in S1 Table. We selected 108 candidate genes with permutation FDRs smaller than 0.05 as the significant candidate genes (see the first 108 genes in S1 Table).

### Gene ontology enrichment analysis of the significant candidate genes

Gene Ontology (GO) is a widely used bioinformatics tool, which represents gene product properties across all species using GO terms [15,25]. Here, we analyzed the 108 significant candidate genes by investigating their GO enrichment terms. This was performed by a functional annotation tool, DAVID (Database for Annotation, Visualization and Integrated Discovery) [26]. The results are shown in Table 1.

**Table 1:** The GO BP enrichment analysis of the 108 significant candidate genes.

Term	Count	FDR
GO:0042127~regulation of cell proliferation	25	3.15E-06
GO:0008284~positive regulation of cell proliferation	17	9.54E-05
GO:0060284~regulation of cell development	12	5.43E-04
GO:0010720~positive regulation of cell development	8	0.0012
GO:0048010~vascular endothelial growth factor receptor signaling pathway	5	0.0015
GO:0044093~positive regulation of molecular function	18	0.0021
GO:0006357~regulation of transcription from RNA polymerase II promoter	20	0.0022
GO:0008285~negative regulation of cell proliferation	14	0.0041
GO:0048729~tissue morphogenesis	10	0.0121
GO:0035270~endocrine system development	7	0.0194
GO:0051960~regulation of nervous system development	10	0.0204
GO:0010604~positive regulation of macromolecule metabolic process	20	0.0236
GO:0051098~regulation of binding	9	0.0289
GO:0045944~positive regulation of transcription from RNA polymerase II promoter	13	0.0305
GO:0051173~positive regulation of nitrogen compound metabolic process	17	0.0313
GO:0007183~SMAD protein complex assembly	4	0.0359
GO:0045927~positive regulation of growth	7	0.0456
GO:0043085~positive regulation of catalytic activity	15	0.0459

The 108 significant candidate genes were significantly enriched in Biological Process (BP) terms related to neuronal cell proliferation and development: GO:0042127 (Regulation of cell proliferation), GO:0008284 (Positive regulation of cell proliferation), GO:0060284 (Regulation of cell development), GO:0010720 (Positive regulation of cell development), GO:0051960 (Regulation of nervous system development) and GO:0035270 (Endocrine system development).

### Analysis of the relationship between several significant candidate genes and the biological process of brain development

In this study, we obtained 108 genes that possibly relate to brain development, among which 16 genes (14.81%, 16/108) were GO inferred genes. These 16 genes are listed in Table 2. In the following paragraphs, previously reported experimental evidence are provided for the expression and functions of these inferred genes in brain development, indicating that our method is effective for the discovery of new candidate genes.

**Table 2:** Detailed information for the 16 significant candidate genes that were also inferred genes.

Ensembl ID	Gene name	Betweenness	Permutation FDR
ENSP00000287934	FZD1	88	0
ENSP00000354607	FZD5	87	0.02
ENSP00000363826	FZD8	88	0
ENSP00000305769	SMAD1	252	0.016
ENSP00000176195	SCT	147	0.048
ENSP00000330633	CNTN2	88	0.004
ENSP00000354478	DLX1	88	0.002
ENSP00000320147	EZH2	88	0.008
ENSP00000354859	DRD2	6	0.016
ENSP00000329623	BCL2	570	0.002
ENSP00000396219	MEF2C	88	0.014
ENSP00000366413	POU4F1	88	0
ENSP00000261349	LRP6	174	0.026
ENSP00000353059	APAF1	250	0.018
ENSP00000237527	GHRH	4	0.02
ENSP00000320180	GHRHR	2	0.03

The Frizzled proteins were identified to be the receptors for the Wnt signaling molecules [27,28]. Wnt proteins are highly expressed in the developing central nervous system and were shown to play essential roles in brain development, adult neurogenesis and brain disorders [3,29,30]. In our study, Frizzled receptors *FZD1*, *FZD5* and *FZD8* were predicted to be involved in brain development, which is consistent with previous observations.

The growth hormone releasing hormone *GHRH* and its receptor *GHRHR* were also predicted to be related to brain development. The expression of both GHRH and GHRHR were detected in the brain [31,32]. GHRHR was highly expressed in the hypothalamus and pituitary, but not in the olfactory bulb, caudate putamen, cerebral cortex, hippocampus, cerebellum or brainstem, suggesting possible sites of GHRH action. GHRH stimulates the release of growth hormone, which has been shown to alter neurogenesis, myelin synthesis and dendritic branching [33].

SMADs are important signaling molecules of cytokine-mediated signaling pathways and are involved in a series of physiological and pathological processes. One of the family members, *SMAD1*, was reported to form a complex with STAT3 (signal transducers and activators of transcription), and the formation process was bridged by the transcriptional coactivator EP300. This complex was involved in the cooperative signaling of LIF (leukemia inhibitory factor) and BMP2 (bone morphogenetic protein-2) and the subsequent induction of astrocytes from neural progenitors [34]. Our study predicted roles for *SMAD1*, *EP300* and other SMAD family members such as *SMAD2* and *SMAD4*.

The *SCT* gene encodes a gastrointestinal peptide secretin, which is widely expressed during mouse embryonic development [35]. Several lines of evidence indicate that the expression of secretin or secretin-like peptides were also present in several developing brain regions such as the cephalic mesenchyme, cerebellar primordium and choroid plexus [35,36,37,38]. Secretin deficient mice demonstrated impairment in synaptic plasticity in the CA1 area of the hippocampus, which implied the potential neuroactive role of secretin in the brain [39].

Among the contactin family, *CNTN1* (also known as contactin) has been extensively investigated for its role in the brain. The GPI-linked neural cell recognition molecule F3/contactin is clustered at the paranodal region during development, a vital site for axoglial interaction. F3/contactin was reported to act as a functional ligand of Notch, and this trans-extracellular interaction triggered gamma-secretase-dependent nuclear translocation of the Notch intracellular domain. The F3/contactin was shown to specifically initiate a Notch/Deltex1 signaling pathway that promoted oligodendrocyte maturation and myelination [40]. Our study predicted a potential role of *CNTN2*, which was expressed on the surface neuronal cells [41] in brain development.

The POU class 4 homeobox 1 protein POU4F1 has been shown to regulate dorsal-root ganglion-sensory neuron specification and axonal projection into the spinal cord [42]. Additionally, high expression of POU4F1 and other POU-homeodomain transcription factors were detected in developing projection neurons within the retina, inner ear and trigeminal ganglion with an established function in controlling cell differentiation and survival [43].

The enhancer of zeste homolog 2 (EZH2) is a member of the Polycomb-group (PcG) family, which is involved in maintaining the transcriptional repressive state of genes over successive cell generations by forming multimeric protein complexes [44]. Because of the central role of EZH2 in epigenetic regulations of cell proliferation and differentiation, misregulation of neural progenitor-cell differentiation during cortical development was observed when EZH2 expression was repressed by the upstream nuclear factor IB (NFIB) [45].

Besides the above-mentioned GO inferred genes, mounting evidence has suggested the potential roles of other significant candidate genes in brain development, such as *CNOT1*, *CNOT2*, *CNOT4*, *CNOT6*, *MED8*, *MED10*, *MED12*, *CDK8*, *LAMA2*, *LAMA4*, *CASP1* and *etc*.

The CNOT subunits form the CCR4-NOT complex, which is a highly conserved global transcriptional regulator. This complex has been implicated in a number of different aspects of mRNA and protein expression, including mRNA degradation, transcription initiation and elongation, ubiquitination and protein modification [46], which is essential in the context of development [47]. Previous studies described the spatiotemporal expression of CNOTs during differentiation of neural stem cells, implying their function in brain development [48].

The mediator complex aids in transcriptional activation through interaction with RNA polymerase II and gene-specific transcription factors. Here, subunits CDK8, MED8, MED10 and MED12 were predicted to be related to brain development. The cyclin-dependent kinase CDK8 served as a partner of MED12 in the mediator complex that functioned in developmental gene regulation [49]. And a recent study unveiled a new role of the mediator complex in epigenetic silencing of neuronal gene expression, which is linked to neuronal development and function [50].

Laminin subunits LAMA2 and LAMA4 were also predicted to be involved in brain development. The extracellular protein laminin is a major component of the basement membrane and mediate the attachment, migration, and organization of cells into tissues during embryonic development. Laminin has been shown to regulate neural precursor cells by enhancing migration, expansion, and differentiation into neurons and astrocytes [51]. Additionally, short laminin peptides were reported to promote in vitro neural stem cell proliferation and survival [52].

The caspases are a family of cysteine-aspartic acid protease, which play crucial roles in programmed cell death [53]. Recent evidence indicated that caspase family also participated in various developmental processes, including the development of thymocyte [54], kidney [55], retina [56], tooth [57], and the central nervous system [58]. Several caspase family members such as caspase 3, 8 and 9 have been shown to regulate the postnatal brain development [59,60], and caspase 1 (*CASP1*) was predicted by our algorithm to be also involved in this process.

Here, we have provided some experimental evidence for eleven candidate genes of their functions in brain development. These results are consistent with our prediction and suggest the effectiveness and efficiency of our algorithm. Other candidate genes also serve as a useful resource for further investigation on brain development and function.

Furthermore, it is well known that aberrant expression of genes with physiological functions often leads to diseases [61]. To understand the potential implication of our candidate genes in brain diseases, we performed hypergeometric enrichment test between the 108 candidate genes and the genes of several well-known brain diseases, including brain tumor, autism, schizophrenia, Alzheimer disease and Parkinson disease. The brain disease gene data were extracted from DisGeNET v2.1 [62] and the results were shown in Table 3. For instance, the Frizzled receptors (*FZD1*, *FZD5* and *FZD8*) may also be involved in the brain carcinogenesis, Alzheimer disease and schizophrenia through disordered Wnt signaling [3,63,64]. The *BCL2* oncogene encodes an integral outer mitochondrial membrane protein which blocks the apoptotic death of cells, and it has been well established that BCL2 promoted carcinogenesis in different tissues [65,66]. Additionally, BCL2 was also suggested to be related to autism as its level significantly decreased in the autistic brain [67]. Our candidate gene set contains another proto-oncogene *MYC* with an established role in several cancer types [68]. Dopamine receptors were reported to be closely related to the development of schizophrenia [69]. Among them, dopamine receptor D2 (*DRD2*) was a candidate gene with potential biological functions during brain development. All these examples indicated a dual character of brain development-related genes that they can function in either physiological or pathological ways.

**Table 3:** The enrichment between the 108 candidate genes and the genes of well-known brain diseases.

Brain disease	Enrichment p value	Overlapped genes
Alzheimer Disease	3.88E-10	MAPT, BCL2, CR1, ABCA7, ENO1, COMT, UCHL1, GFAP, DRD2, LRP6, GNB3, C4B, MYC, PTH1R, KDR, IGFBP6, GHRHR, GHRH, EFNA3, GPC1, STUB1, ATF4, NLRP1, CASP1, DCP1B, CNTF, GPI, MED12, VIM, EP300, HDAC4, NCL, FKBP1A, FZD5, FOXM1, EIF2AK3
Glioma	6.38E-08	BAD, GFAP, KDR, NRP1, BCL2, NF2, VIM, AURKB, MYC, FOXM1, SMAD2, RICTOR, LAMA4, EZH2, PKM, FIGF, CDC25B, PTHLH, CNTF, EFNB2, EPHA4, SNRPE, STUB1, GPC1, EP300, CNTN2
Schizophrenia	1.96E-05	COMT, DRD2, KDR, LAMA2, GNB3, CNTF, MED12, EP300, ATF4, GFAP, NRP1, HDAC4, DLX1, MAPT, EFNB2, GPC1, ABCA7, ATP5H, STUB1, VIM, CASP1, NUP98, PTHLH, SRGAP1
Parkinson Disease	0.000573	DRD2, MAPT, GFAP, UCHL1, COMT, BAD, EPHA4, APAF1, MEF2C, NCL, BCL2, FKBP1A, CR1, NLRP1, STUB1, C4B
Autistic Disorder	0.00215	C4B, COMT, DLX1, SCT, BCL2, MEF2C, DRD2, CYP21A2, MED12, RYR1, ABCA7

### Conclusions

This study utilized the shortest path algorithm to discover new candidate genes that are related to brain development. Furthermore, a randomization test was adopted to filter the positive discoveries. Among the final obtained genes, several have direct evidence for their expression and functions in the brain, whereas several others may play potential roles in brain development. The findings in this study provide new insights for the discovery of novel genes related to brain development, thereby promoting the comprehension of the overall developmental process. The software is available upon the request.

## Supporting Information

S1 File 94 human genes related to brain development, which are with experimental evidence from Gene Ontology (GO:0007420). (PDF)

S2 File 516 human genes related to brain development, which are with evidence, instead of experimental evidence, from Gene Ontology (GO:0007420). (PDF)

S1 Table 382 candidate genes for brain development and their betweenness and permutation FDRs. (PDF)
