# Supplementary material for: Discovery of New Candidate Genes Related to Brain Development Using Protein Interaction Information
Source: PLoS One. 2015 Jan 30;10(1):e0118003. doi: 10.1371/journal.pone.0118003 (PMC4311913; doi:10.1371/journal.pone.0118003)
Supplement: S1 File — (PDF) [file pone.0118003.s001.pdf]

S1 File. 94 human genes related to brain development, which are with experimental evidence from Gene Ontology (GO:0007420)

SHROOM4
ROBO1
NDE1
GPR56
NPY
ZNF148
SYNGR3
SRGAP2C
NDUFS4
ROBO2
ZIC1
BASP1
SALL1
CTNS
LAMB1
SMARCA1
CNTNAP2
PADI2
GNB4
TTBK1
BPTF
POTEE
NDRG2
DYNLL1
WDR62
EOMES
ATP5J
SEMA3A
MAG
SEC16A
MBP
PTF1A
WNT7B
CNP
HSPA5
WNT2B
G6PD
RHOA
S100A1
COX6B1
COL4A1

LRRK2
WNT7A
SZT2
ZNF335
H2BFS
PTPN11
BMP4
BMP2
SUDS3
SYPL2
ENO3
YWHAQ
MAOB
LDHA
ASCL1
CKB
MAPKAP1
NOG
NKX2-1
SLC6A4
FOXP2
CCDC14
SLIT2
AQP1
GLUD1
PROX1
CDC42
DSCAML1
H2AFY2
PAFAH1B1
INA
ACTB
RAD1
ABCB6
VCX3A
YWHAH
FGF9
PLP1
GSK3B
SIRT2
CASP5
RAB3GAP1
ZNF430
CLN5

NDUFS3
PPT1
ATP5F1
SLIT3
SLIT1
SOX2
YWHAE
CALM1
NIPBL
