# Supplementary material for: Discovery of New Candidate Genes Related to Brain Development Using Protein Interaction Information
Source: PLoS One. 2015 Jan 30;10(1):e0118003. doi: 10.1371/journal.pone.0118003 (PMC4311913; doi:10.1371/journal.pone.0118003)
Supplement: S2 File — (PDF) [file pone.0118003.s002.pdf]

S2 File. 516 human genes related to brain development, which are with evidence, instead of experimental evidence, from Gene Ontology (GO:0007420)

ABR
AK2
AK4
AK7
AK8
APP
ARX
ATM
AXL
BAX
BCR
BID
BOK
CBS
CD9
CRH
CSK
DCT
DCX
EGF
EN1
EN2
FYN
GSC
HTT
ID2
ID4
MDK
MET
NES
NF1
OTP
RAX
RYK
SCT
SHH
SKI
SMO
SRC
SRF
SRR

TH
TNR
AARS
AATK
ACAT1
ACSL3
ADCYAP1
AFF2
AGTPBP1
AGTR2
AHI1
ALDH1A2
ALDH1A3
ALX1
APAF1
APLP1
APLP2
APOD
ARCN1
ARF4
ARID1A
ARL13B
ARRB2
ASPM
ATF5
ATG7
ATIC
ATOH1
ATP2B2
ATP7A
ATRN
ATRX
ATXN2
AVPR1A
AVPR2
AXIN1
BAG3
BAG6
BAK1
BARHL1
BBS2
BBS4
BBS7
BCAN

BCL11B
BCL2
BCL2L11
BDH1
BHLHE22
BMPR1A
BMPR2
BRCA2
BTBD3
BTG2
C2CD3
C5AR1
CA10
CACNA1A
CADM1
CADM2
CASP2
CBLN1
CCDC85C
CCKAR
CCNG1
CDH1
CDH22
CDK5
CDK5R1
CDK5R2
CDK6
CDON
CELSR2
CEND1
CEP120
CEP290
CHD5
CHRD
CHRNB2
CITED1
CMA1
CNTN1
CNTN2
CNTN4
COL3A1
COX17
CREB1
CRHR1

CTNNA2
CTNNB1
CTNND1
CTTNBP2
CX3CR1
CXCL12
CXCR2
CXCR4
CYP11A1
CYP17A1
DAB1
DAB2IP
DCLK1
DDIT4
DFFB
DICER1
DISC1
DIXDC1
DKK1
DLC1
DLX1
DLX2
DMBX1
DPCD
DPYSL2
DRAXIN
DRD1
DRD2
DUOX2
DVL2
DYNC2H1
E2F1
ECE2
EFNA2
EGFR
EGR2
EMX1
EMX2
EPHA5
EPHA7
EPHB2
EPHB3
EPOR
ERBB4

ESR2
ETS1
EVI1
EXT1
EZH2
FABP7
FAIM2
FAT4
FEZF1
FEZF2
FGF10
FGF13
FGF2
FGF8
FGFR1
FGFR2
FGFR3
FOXA2
FOXB1
FOXC1
FOXG1
FOXJ1
FOXL1
FPGS
FRS2
FRZB
FZD1
FZD10
FZD2
FZD3
FZD4
FZD5
FZD6
FZD7
FZD8
FZD9
GABRA5
GART
GAS1
GATA2
GBX2
GDF7
GDPD5
GHRH

GHRHR
GLI1
GLI2
GLI3
GNAO1
GNAQ
GNG12
GNPAT
GRID2
GRIN1
GSX1
GSX2
H3F3A
H3F3B
HAP1
HDAC2
HERC1
HES1
HES3
HES5
HESX1
HHEX
HIF1A
HMGA2
HMGCS1
HMX2
HMX3
HNF1B
HNMT
HOOK3
HOXA1
HOXA2
HOXB1
HOXB2
HOXB3
HPRT1
HRH3
HSPG2
HTR5A
HTR6
HTRA2
IFT88
IGF1
IGF1R

IKZF1
IRS2
ISL1
ITGA8
ITGB1
KAT2A
KCNK3
KDM1A
KDM2B
KDM7A
KIF27
KIRREL3
KLHL1
KLHL17
KNDC1
LDB1
LEF1
LHX1
LHX2
LHX3
LHX5
LHX6
LHX8
LMX1A
LMX1B
LRP2
LRP6
LRP8
MACROD2
MAFB
MAP1S
MAS1
MBOAT7
MDGA1
MECOM
MECP2
MED1
MEF2A
MEF2C
MEN1
MKKS
MNAT1
MSX1
MTPN

MYH10
MYO16
NAGLU
NAPA
NCOA1
NCOA6
NCOR1
NCOR2
NDEL1
NDST1
NEURL1
NEUROD1
NEUROD2
NEUROD6
NEUROG2
NEUROG3
NFIB
NFIX
NKX2-2
NKX2-6
NLGN4X
NME1
NME5
NME7
NNAT
NODAL
NOTCH1
NOTCH3
NR0B1
NR2C2
NR2E1
NR2F1
NR2F2
NR4A2
NR4A3
NRXN1
NTF3
NTRK2
NUMB
NUMBL
OGDH
OLIG2
OTX1
OTX2

OXCT1
OXTR
P2RY1
PAX2
PAX6
PCDH18
PCDH9
PCM1
PCNT
PEX13
PEX5
PFDN1
PGAP1
PHF8
PHGDH
PHLDA1
PHOX2A
PHOX2B
PITPNM1
PITX1
PITX2
PITX3
PLCB1
PLXNA2
PLXNA3
PLXNA4
PLXNB2
POMK
POU1F1
POU3F1
POU3F2
POU3F3
POU3F4
POU4F1
POU6F1
PRDM8
PRKDC
PRKG1
PROP1
PSEN1
PSEN2
PTCH1
PTEN
PTPRG

PTPRS
PYGO2
RAB18
RAC1
RALDH2
RAPGEF2
RARB
RBFOX2
RBPJ
RELN
RFX4
RORA
RRM1
RTN4
S1PR1
SALL3
SCN5A
SDF4
SEMA4C
SEMA5A
SEMA6D
SEPP1
SEPT4
SETD2
SEZ6
SEZ6L
SEZ6L2
SFRP1
SFRP2
SFRP4
SFRP5
SHANK3
SHARPIN
SHROOM2
SIX3
SKOR2
SLC1A2
SLC23A1
SLC4A7
SLC6A11
SLC6A3
SLC7A11
SLC8A3
SLITRK5

SMAD1
SMAD9
SMARCA4
SNPH
SOX1
SOX3
SPEF2
SPHK1
SPHK2
SPTBN2
SRGAP2
SSBP3
SSTR1
SSTR2
SSTR3
SSTR4
STAR
STIL
STK36
TACC1
TACC2
TACC3
TAL2
TBR1
TBX19
TBX3
TCF7
TCF7L1
TCF7L2
TCTN1
TFAP2A
TFAP2C
TFAP2D
TGFBR2
TMEM57
TOP2B
TP73
TRNP1
TSC1
TSKU
TSPAN2
TTC8
TULP3
TWSG1

TYRO3
UBE3A
UCHL5
ULK1
ULK4
UNC5C
UNCX
UQCRQ
UTP3
VAX1
VAX2
WNT1
WNT3A
WNT4
WNT5A
XRCC1
XRCC5
XRCC6
ZBTB18
ZEB1
ZEB2
ZFHX3
ZIC2
ZIC5
ARHGAP35
ATP6V0D1
CDK5RAP1
CDK5RAP2
CDK5RAP3
MAPK8IP3
PAFAH1B2
PAFAH1B3
RPGRIP1L
SERPINE2
DKFZp686L0365
