# Supplementary material for: Discovery of New Candidate Genes Related to Brain Development Using Protein Interaction Information
Source: PLoS One. 2015 Jan 30;10(1):e0118003. doi: 10.1371/journal.pone.0118003 (PMC4311913; doi:10.1371/journal.pone.0118003)
Supplement: S1 Table — (PDF) [file pone.0118003.s003.pdf]

**S1 Table.** 382 candidate genes for brain development and their betweenness and permutation FDRs.

| Ensembl ID of genes | Gene name | Betweenness | Permutation FDR |
|---|---|---|---|
| ENSP00000241651 | MYOG | 88 | 0 |
| ENSP00000245960 | CDC25B | 38 | 0 |
| ENSP00000247182 | SIX1 | 88 | 0 |
| ENSP00000248150 | GNG13 | 84 | 0 |
| ENSP00000253408 | GFAP | 88 | 0 |
| ENSP00000261937 | FLT4 | 88 | 0 |
| ENSP00000261951 | CNOT6 | 88 | 0 |
| ENSP00000263094 | ABCA7 | 88 | 0 |
| ENSP00000267859 | BNIP2 | 133 | 0 |
| ENSP00000269280 | NLRP1 | 88 | 0 |
| ENSP00000282588 | ITGA1 | 88 | 0 |
| ENSP00000284440 | UCHL1 | 88 | 0 |
| ENSP00000287934 | FZD1 | 88 | 0 |
| ENSP00000290663 | MED8 | 88 | 0 |
| ENSP00000300619 | ZNF91 | 88 | 0 |
| ENSP00000301464 | IGFBP6 | 88 | 0 |
| ENSP00000318195 | NCL | 174 | 0 |
| ENSP00000344666 | NF2 | 88 | 0 |
| ENSP00000347198 | SRGAP1 | 339 | 0 |

| | | | |
|---|---|---|---|
| ENSP00000351832 | RAB3GAP2 | 88 | 0 |
| ENSP00000357393 | EFNA3 | 88 | 0 |
| ENSP00000359151 | DBT | 88 | 0 |
| ENSP00000362592 | RBBP4 | 179 | 0 |
| ENSP00000363826 | FZD8 | 88 | 0 |
| ENSP00000366413 | POU4F1 | 88 | 0 |
| ENSP00000370445 | HTN3 | 88 | 0 |
| ENSP00000175506 | ASNS | 88 | 0.002 |
| ENSP00000224237 | VIM | 88 | 0.002 |
| ENSP00000229195 | CNOT2 | 88 | 0.002 |
| ENSP00000311360 | RAD9A | 88 | 0.002 |
| ENSP00000329623 | BCL2 | 570 | 0.002 |
| ENSP00000349959 | RICTOR | 88 | 0.002 |
| ENSP00000354478 | DLX1 | 88 | 0.002 |
| ENSP00000354720 | SMC3 | 88 | 0.002 |
| ENSP00000373715 | DCP2 | 97 | 0.002 |
| ENSP00000410076 | CASP1 | 88 | 0.002 |
| ENSP00000260810 | TOPBP1 | 88 | 0.004 |
| ENSP00000296930 | NPM1 | 174 | 0.004 |
| ENSP00000307235 | EIF2AK3 | 88 | 0.004 |
| ENSP00000320949 | CNOT1 | 88 | 0.004 |
| ENSP00000330633 | CNTN2 | 88 | 0.004 |

| | | | |
|---|---|---|---|
| ENSP00000340820 | MAPT | 94 | 0.004 |
| ENSP00000341551 | SMAD4 | 415 | 0.004 |
| ENSP00000354511 | COMT | 88 | 0.004 |
| ENSP00000354673 | CNOT4 | 88 | 0.004 |
| ENSP00000370421 | PDX1 | 88 | 0.004 |
| ENSP00000381717 | UBE2D2 | 88 | 0.004 |
| ENSP00000403721 | CYP21A2 | 88 | 0.004 |
| ENSP00000255764 | MED10 | 88 | 0.006 |
| ENSP00000262030 | ATP5B | 168 | 0.006 |
| ENSP00000263923 | KDR | 98 | 0.006 |
| ENSP00000280193 | VEGFC | 62 | 0.006 |
| ENSP00000312122 | SEC13 | 88 | 0.006 |
| ENSP00000355370 | CNTF | 88 | 0.006 |
| ENSP00000280665 | DCP1B | 42 | 0.008 |
| ENSP00000301587 | ATP5H | 6 | 0.008 |
| ENSP00000320147 | EZH2 | 88 | 0.008 |
| ENSP00000321999 | PTH1R | 15 | 0.008 |
| ENSP00000219548 | STUB1 | 3 | 0.01 |
| ENSP00000265371 | NRP1 | 88 | 0.01 |
| ENSP00000315774 | NDUFS8 | 1 | 0.01 |
| ENSP00000317159 | CYC1 | 252 | 0.01 |
| ENSP00000352608 | RYR1 | 175 | 0.01 |

| | | | |
|---|---|---|---|
| ENSP00000354554 | MT-CYB | 88 | 0.01 |
| ENSP00000354876 | MT-CO2 | 88 | 0.01 |
| ENSP00000307786 | CYCS | 250 | 0.012 |
| ENSP00000230538 | LAMA4 | 64 | 0.014 |
| ENSP00000336790 | ATF4 | 168 | 0.014 |
| ENSP00000396219 | MEF2C | 88 | 0.014 |
| ENSP00000281821 | EPHA4 | 88 | 0.016 |
| ENSP00000305769 | SMAD1 | 252 | 0.016 |
| ENSP00000354859 | DRD2 | 6 | 0.016 |
| ENSP00000371138 | FKBP1A | 186 | 0.016 |
| ENSP00000264606 | HDAC4 | 9 | 0.018 |
| ENSP00000353059 | APAF1 | 250 | 0.018 |
| ENSP00000237527 | GHRH | 4 | 0.02 |
| ENSP00000263253 | EP300 | 740 | 0.02 |
| ENSP00000297904 | FIGF | 3 | 0.02 |
| ENSP00000309103 | BAD | 160 | 0.02 |
| ENSP00000320171 | PKM | 69 | 0.02 |
| ENSP00000354607 | FZD5 | 87 | 0.02 |
| ENSP00000367207 | MYC | 289 | 0.022 |
| ENSP00000370938 | CDK8 | 88 | 0.022 |
| ENSP00000262160 | SMAD2 | 358 | 0.024 |
| ENSP00000261349 | LRP6 | 174 | 0.026 |

| | | | |
|---|---|---|---|
| ENSP00000264951 | XRN1 | 195 | 0.028 |
| ENSP00000320180 | GHRHR | 2 | 0.03 |
| ENSP00000356016 | CR1 | 88 | 0.03 |
| ENSP00000415941 | C4B | 88 | 0.03 |
| ENSP00000234590 | ENO1 | 68 | 0.032 |
| ENSP00000342307 | FOXM1 | 64 | 0.032 |
| ENSP00000348877 | GPI | 88 | 0.032 |
| ENSP00000363193 | MED12 | 1 | 0.032 |
| ENSP00000316032 | NUP98 | 88 | 0.034 |
| ENSP00000379213 | PTHLH | 5 | 0.034 |
| ENSP00000229264 | GNB3 | 84 | 0.038 |
| ENSP00000297338 | RAD21 | 5 | 0.038 |
| ENSP00000223023 | WASL | 120 | 0.04 |
| ENSP00000245323 | EFNB2 | 88 | 0.04 |
| ENSP00000264039 | GPC1 | 1 | 0.04 |
| ENSP00000309871 | RACGAP1 | 21 | 0.044 |
| ENSP00000313950 | AURKB | 88 | 0.044 |
| ENSP00000323421 | SMC1A | 83 | 0.044 |
| ENSP00000353622 | SIN3A | 88 | 0.046 |
| ENSP00000176195 | SCT | 147 | 0.048 |
| ENSP00000395498 | PAX8 | 86 | 0.048 |
| ENSP00000400365 | LAMA2 | 24 | 0.048 |

| | | | |
|---|---|---|---|
| ENSP00000400591 | SNRPE | 207 | 0.048 |
| ENSP00000264634 | WNT5A | 87 | 0.05 |
| ENSP00000295731 | IHH | 5 | 0.05 |
| ENSP00000352264 | CD2AP | 2 | 0.05 |
| ENSP00000297261 | SHH | 91 | 0.054 |
| ENSP00000361125 | VEGFA | 122 | 0.054 |
| ENSP00000318351 | BCKDHB | 88 | 0.056 |
| ENSP00000227378 | HSPA8 | 95 | 0.058 |
| ENSP00000270349 | SLC6A3 | 6 | 0.058 |
| ENSP00000228872 | CDKN1B | 134 | 0.06 |
| ENSP00000262187 | RHEB | 3 | 0.06 |
| ENSP00000271555 | MEF2D | 10 | 0.06 |
| ENSP00000236850 | APOA1 | 88 | 0.064 |
| ENSP00000250448 | FOXA1 | 1 | 0.064 |
| ENSP00000256383 | EIF2S1 | 88 | 0.064 |
| ENSP00000357625 | BNIP3 | 88 | 0.064 |
| ENSP00000364133 | TGFBR1 | 88 | 0.064 |
| ENSP00000378338 | GIT1 | 19 | 0.064 |
| ENSP00000263360 | EED | 4 | 0.066 |
| ENSP00000261023 | ITGAV | 54 | 0.068 |
| ENSP00000007722 | ITGA3 | 6 | 0.07 |
| ENSP00000227667 | APOC3 | 54 | 0.07 |

| | | | |
|---|---|---|---|
| ENSP00000245907 | C3 | 88 | 0.07 |
| ENSP00000252102 | NDUFA2 | 174 | 0.07 |
| ENSP00000268182 | IQGAP1 | 154 | 0.07 |
| ENSP00000222330 | GSK3A | 6 | 0.074 |
| ENSP00000244007 | PLCG1 | 113 | 0.074 |
| ENSP00000332353 | PTCH1 | 5 | 0.074 |
| ENSP00000344352 | ATF3 | 167 | 0.074 |
| ENSP00000380280 | FGFR1 | 152 | 0.076 |
| ENSP00000247170 | DAAM1 | 1 | 0.08 |
| ENSP00000319664 | NUDC | 141 | 0.082 |
| ENSP00000392423 | RELN | 8 | 0.082 |
| ENSP00000302665 | IGF1 | 88 | 0.084 |
| ENSP00000356024 | CR2 | 88 | 0.084 |
| ENSP00000278616 | ATM | 83 | 0.086 |
| ENSP00000338072 | AVPR2 | 85 | 0.086 |
| ENSP00000355537 | ACTN2 | 89 | 0.086 |
| ENSP00000243050 | NR4A1 | 135 | 0.088 |
| ENSP00000351407 | ARNT | 69 | 0.09 |
| ENSP00000278568 | PAK1 | 50 | 0.094 |
| ENSP00000388107 | UBA52 | 88 | 0.094 |
| ENSP00000302269 | VAV1 | 132 | 0.096 |
| ENSP00000364893 | ARHGEF7 | 56 | 0.096 |

| | | | |
|---|---|---|---|
| ENSP00000282397 | FLT1 | 3 | 0.098 |
| ENSP00000298552 | TSC1 | 51 | 0.098 |
| ENSP00000338799 | IL6ST | 89 | 0.098 |
| ENSP00000254976 | SNAP25 | 88 | 0.104 |
| ENSP00000344822 | S100A13 | 4 | 0.104 |
| ENSP00000364094 | ITGB1 | 109 | 0.106 |
| ENSP00000303634 | LRP8 | 4 | 0.108 |
| ENSP00000351665 | CLIP1 | 14 | 0.108 |
| ENSP00000365891 | WAS | 22 | 0.11 |
| ENSP00000256442 | CCNB1 | 52 | 0.112 |
| ENSP00000280357 | IL18 | 88 | 0.112 |
| ENSP00000379933 | TPI1 | 87 | 0.112 |
| ENSP00000309597 | MAP3K11 | 5 | 0.114 |
| ENSP00000354687 | MT-ND1 | 2 | 0.114 |
| ENSP00000363708 | BMPR2 | 2 | 0.116 |
| ENSP00000261205 | SYT1 | 4 | 0.12 |
| ENSP00000414006 | LSM2 | 19 | 0.12 |
| ENSP00000241014 | MAPK8IP1 | 5 | 0.122 |
| ENSP00000260402 | PLCB2 | 4 | 0.122 |
| ENSP00000264708 | POMC | 160 | 0.122 |
| ENSP00000299293 | FRS2 | 78 | 0.122 |
| ENSP00000345571 | E2F1 | 96 | 0.122 |

| | | | |
|---|---|---|---|
| ENSP00000358622 | IKBKG | 88 | 0.124 |
| ENSP00000287820 | PPARG | 86 | 0.126 |
| ENSP00000310491 | ARHGAP1 | 16 | 0.126 |
| ENSP00000338018 | HIF1A | 261 | 0.126 |
| ENSP00000379204 | BMP7 | 62 | 0.128 |
| ENSP00000360141 | GNAS | 84 | 0.13 |
| ENSP00000256897 | CCNH | 23 | 0.134 |
| ENSP00000301633 | BIRC5 | 72 | 0.138 |
| ENSP00000348708 | UPF2 | 49 | 0.138 |
| ENSP00000360286 | RAE1 | 36 | 0.138 |
| ENSP00000300651 | MED1 | 64 | 0.14 |
| ENSP00000215832 | MAPK1 | 199 | 0.142 |
| ENSP00000312987 | HNF4A | 6 | 0.142 |
| ENSP00000298171 | TSHR | 1 | 0.144 |
| ENSP00000362413 | PGK1 | 87 | 0.144 |
| ENSP00000358918 | SUFU | 1 | 0.148 |
| ENSP00000229022 | VDR | 63 | 0.15 |
| ENSP00000229239 | GAPDH | 87 | 0.15 |
| ENSP00000264867 | PPARGC1A | 66 | 0.152 |
| ENSP00000363019 | UBE2D1 | 14 | 0.154 |
| ENSP00000264246 | CD80 | 4 | 0.158 |
| ENSP00000297518 | CDK5 | 10 | 0.16 |

| | | | |
|---|---|---|---|
| ENSP00000354923 | DMD | 2 | 0.16 |
| ENSP00000302160 | LSM3 | 7 | 0.162 |
| ENSP00000313419 | CD19 | 88 | 0.166 |
| ENSP00000363804 | KLF4 | 6 | 0.166 |
| ENSP00000310596 | LSM1 | 41 | 0.168 |
| ENSP00000216911 | AURKA | 29 | 0.174 |
| ENSP00000258682 | CAMK2B | 124 | 0.174 |
| ENSP00000333001 | RBM8A | 8 | 0.174 |
| ENSP00000399968 | NCOA2 | 80 | 0.176 |
| ENSP00000267163 | RB1 | 120 | 0.178 |
| ENSP00000263754 | KAT2B | 30 | 0.18 |
| ENSP00000282091 | PTH | 81 | 0.182 |
| ENSP00000317714 | STX4 | 3 | 0.184 |
| ENSP00000332049 | CD86 | 12 | 0.19 |
| ENSP00000363827 | HSPG2 | 1 | 0.19 |
| ENSP00000261396 | NUP133 | 52 | 0.192 |
| ENSP00000276201 | UPF3B | 8 | 0.192 |
| ENSP00000368169 | DVL1 | 86 | 0.194 |
| ENSP00000278916 | CHEK1 | 100 | 0.198 |
| ENSP00000300161 | YWHAB | 1 | 0.198 |
| ENSP00000354621 | SMURF1 | 7 | 0.206 |
| ENSP00000262077 | NUP153 | 52 | 0.208 |

| | | | |
|---|---|---|---|
| ENSP00000262320 | AXIN1 | 69 | 0.208 |
| ENSP00000324890 | CD28 | 16 | 0.208 |
| ENSP00000338345 | SNCA | 201 | 0.208 |
| ENSP00000354586 | GLI2 | 6 | 0.208 |
| ENSP00000233242 | APOB | 15 | 0.212 |
| ENSP00000257408 | KLB | 1 | 0.214 |
| ENSP00000290158 | KPNB1 | 27 | 0.218 |
| ENSP00000369050 | CYP1A1 | 84 | 0.222 |
| ENSP00000338934 | EZR | 4 | 0.224 |
| ENSP00000279593 | GRIN2B | 95 | 0.244 |
| ENSP00000237837 | FGF23 | 1 | 0.246 |
| ENSP00000331746 | CALCA | 61 | 0.246 |
| ENSP00000302564 | BCL2L1 | 8 | 0.25 |
| ENSP00000320935 | SLC2A4 | 3 | 0.25 |
| ENSP00000304895 | IRS1 | 136 | 0.258 |
| ENSP00000321797 | FGF8 | 70 | 0.258 |
| ENSP00000354558 | MTOR | 123 | 0.258 |
| ENSP00000292408 | FGFR4 | 2 | 0.266 |
| ENSP00000229135 | IFNG | 73 | 0.272 |
| ENSP00000333982 | NDEL1 | 10 | 0.272 |
| ENSP00000363868 | ABCA1 | 13 | 0.278 |
| ENSP00000352561 | CALCR | 61 | 0.28 |

| | | | |
|---|---|---|---|
| ENSP00000361275 | PLK3 | 36 | 0.28 |
| ENSP00000252444 | LDLR | 15 | 0.282 |
| ENSP00000312435 | DAG1 | 24 | 0.284 |
| ENSP00000354791 | DCTN1 | 14 | 0.284 |
| ENSP00000262613 | SLC9A3R1 | 175 | 0.288 |
| ENSP00000312995 | CLSPN | 19 | 0.288 |
| ENSP00000177694 | TBX21 | 9 | 0.298 |
| ENSP00000332973 | SMAD3 | 47 | 0.304 |
| ENSP00000312652 | LEP | 177 | 0.306 |
| ENSP00000294172 | NXF1 | 41 | 0.308 |
| ENSP00000267996 | TPM1 | 19 | 0.31 |
| ENSP00000270202 | AKT1 | 437 | 0.316 |
| ENSP00000325690 | CARM1 | 7 | 0.326 |
| ENSP00000212015 | SIRT1 | 66 | 0.328 |
| ENSP00000352400 | NUP214 | 38 | 0.328 |
| ENSP00000250003 | MYOD1 | 82 | 0.33 |
| ENSP00000368880 | FOXO1 | 3 | 0.33 |
| ENSP00000387662 | GCG | 151 | 0.332 |
| ENSP00000419692 | RXRA | 21 | 0.344 |
| ENSP00000348986 | INS-IGF2 | 122 | 0.346 |
| ENSP00000242057 | AHR | 15 | 0.36 |
| ENSP00000269305 | TP53 | 1340 | 0.362 |

| | | | |
|---|---|---|---|
| ENSP00000219476 | TSC2 | 25 | 0.366 |
| ENSP00000330237 | CASP9 | 1 | 0.368 |
| ENSP00000256443 | CDK7 | 14 | 0.37 |
| ENSP00000339527 | FOXO3 | 14 | 0.37 |
| ENSP00000302486 | MAP2K1 | 14 | 0.376 |
| ENSP00000356713 | IFNGR1 | 8 | 0.376 |
| ENSP00000313829 | KHDRBS1 | 1 | 0.382 |
| ENSP00000344115 | CDH5 | 6 | 0.384 |
| ENSP00000283635 | CD8A | 88 | 0.386 |
| ENSP00000380921 | SH3KBP1 | 30 | 0.39 |
| ENSP00000303830 | INSR | 24 | 0.392 |
| ENSP00000365016 | IRS2 | 3 | 0.392 |
| ENSP00000226218 | SEBOX | 1 | 0.398 |
| ENSP00000261799 | PDGFRB | 130 | 0.4 |
| ENSP00000362649 | HDAC1 | 156 | 0.4 |
| ENSP00000321656 | CDC25C | 145 | 0.402 |
| ENSP00000346300 | CRKL | 8 | 0.402 |
| ENSP00000338548 | FGF1 | 4 | 0.404 |
| ENSP00000380227 | ITGA4 | 90 | 0.414 |
| ENSP00000387699 | CREB1 | 118 | 0.414 |
| ENSP00000398597 | EXOSC6 | 4 | 0.414 |
| ENSP00000274255 | SKP2 | 11 | 0.418 |

| | | | |
|---|---|---|---|
| ENSP00000300093 | PLK1 | 143 | 0.426 |
| ENSP00000361626 | YBX1 | 113 | 0.43 |
| ENSP00000254227 | NR0B2 | 8 | 0.434 |
| ENSP00000358997 | IRAK1 | 12 | 0.442 |
| ENSP00000264033 | CBL | 150 | 0.444 |
| ENSP00000302967 | HDAC3 | 2 | 0.444 |
| ENSP00000357656 | FYN | 100 | 0.444 |
| ENSP00000211998 | VCL | 16 | 0.45 |
| ENSP00000316460 | FYB | 2 | 0.452 |
| ENSP00000306512 | IL8 | 3 | 0.458 |
| ENSP00000353483 | MAPK8 | 134 | 0.458 |
| ENSP00000340691 | EIF4EBP1 | 2 | 0.46 |
| ENSP00000359424 | CHUK | 14 | 0.46 |
| ENSP00000268035 | IGF1R | 29 | 0.462 |
| ENSP00000343204 | JAK1 | 8 | 0.464 |
| ENSP00000299421 | ILK | 14 | 0.466 |
| ENSP00000216160 | TAB1 | 1 | 0.47 |
| ENSP00000347858 | XIAP | 1 | 0.47 |
| ENSP00000252486 | APOE | 69 | 0.472 |
| ENSP00000274376 | RASA1 | 4 | 0.472 |
| ENSP00000329357 | SP1 | 106 | 0.474 |
| ENSP00000274026 | CCNA2 | 48 | 0.478 |

| | | | |
|---|---|---|---|
| ENSP00000280892 | EIF4E | 7 | 0.478 |
| ENSP00000341189 | PTK2 | 78 | 0.48 |
| ENSP00000264972 | ZAP70 | 3 | 0.484 |
| ENSP00000348551 | NCOR2 | 1 | 0.488 |
| ENSP00000354394 | STAT1 | 64 | 0.488 |
| ENSP00000348461 | RAC1 | 248 | 0.492 |
| ENSP00000266970 | CDK2 | 39 | 0.498 |
| ENSP00000252622 | LSM7 | 9 | 0.5 |
| ENSP00000228307 | PXN | 102 | 0.502 |
| ENSP00000355865 | PARK2 | 100 | 0.502 |
| ENSP00000274335 | PIK3R1 | 9 | 0.504 |
| ENSP00000351273 | CASP8 | 4 | 0.504 |
| ENSP00000283195 | RANBP2 | 27 | 0.506 |
| ENSP00000251849 | RAF1 | 53 | 0.522 |
| ENSP00000221494 | SF3A2 | 223 | 0.54 |
| ENSP00000215829 | SNRPD3 | 16 | 0.544 |
| ENSP00000348827 | THRB | 1 | 0.544 |
| ENSP00000262158 | SMAD7 | 2 | 0.546 |
| ENSP00000309503 | YWHAZ | 36 | 0.576 |
| ENSP00000264657 | STAT3 | 333 | 0.58 |
| ENSP00000226730 | IL2 | 101 | 0.582 |
| ENSP00000216797 | NFKBIA | 39 | 0.586 |

| | | | |
|---|---|---|---|
| ENSP00000326804 | CUL1 | 7 | 0.586 |
| ENSP00000263341 | IL1B | 15 | 0.588 |
| ENSP00000356346 | PTPRC | 18 | 0.59 |
| ENSP00000263967 | PIK3CA | 4 | 0.594 |
| ENSP00000257904 | CDK4 | 2 | 0.596 |
| ENSP00000003084 | CFTR | 85 | 0.6 |
| ENSP00000330393 | LEPR | 6 | 0.6 |
| ENSP00000361021 | PTEN | 55 | 0.608 |
| ENSP00000221930 | TGFB1 | 29 | 0.61 |
| ENSP00000304903 | CD2BP2 | 110 | 0.612 |
| ENSP00000269321 | ARHGDIA | 77 | 0.614 |
| ENSP00000222812 | STX1A | 81 | 0.618 |
| ENSP00000339007 | GRB2 | 394 | 0.634 |
| ENSP00000358490 | CD2 | 110 | 0.64 |
| ENSP00000348577 | RANGAP1 | 29 | 0.644 |
| ENSP00000346839 | FN1 | 27 | 0.648 |
| ENSP00000288986 | NCK1 | 5 | 0.668 |
| ENSP00000162330 | BCAR1 | 14 | 0.67 |
| ENSP00000396127 | RAN | 29 | 0.678 |
| ENSP00000360683 | PTPN1 | 10 | 0.68 |
| ENSP00000360266 | JUN | 46 | 0.688 |
| ENSP00000300574 | CRK | 11 | 0.69 |

| | | | |
|---|---|---|---|
| ENSP00000249636 | PIAS1 | 4 | 0.692 |
| ENSP00000255465 | CCNA1 | 10 | 0.7 |
| ENSP00000046794 | LCP2 | 92 | 0.71 |
| ENSP00000259808 | RIPK1 | 4 | 0.718 |
| ENSP00000335153 | HSP90AA1 | 26 | 0.72 |
| ENSP00000339151 | IKBKB | 2 | 0.734 |
| ENSP00000337825 | LCK | 132 | 0.74 |
| ENSP00000324897 | UBE2I | 50 | 0.748 |
| ENSP00000206249 | ESR1 | 133 | 0.762 |
| ENSP00000376076 | SUMO1 | 6 | 0.778 |
| ENSP00000011653 | CD4 | 10 | 0.786 |
| ENSP00000309845 | HRAS | 14 | 0.788 |
| ENSP00000284981 | APP | 61 | 0.792 |
| ENSP00000372023 | CHEK2 | 23 | 0.804 |
| ENSP00000384273 | RELA | 47 | 0.808 |
| ENSP00000363822 | AR | 10 | 0.81 |
| ENSP00000384675 | SOS1 | 8 | 0.814 |
| ENSP00000326366 | PSEN1 | 60 | 0.826 |
| ENSP00000401303 | SHC1 | 25 | 0.828 |
| ENSP00000350283 | BRCA1 | 23 | 0.842 |
| ENSP00000350941 | SRC | 129 | 0.842 |
| ENSP00000227507 | CCND1 | 58 | 0.864 |

| | | | |
|---|---|---|---|
| ENSP00000371067 | JAK2 | 21 | 0.884 |
| ENSP00000320940 | NCOA1 | 1 | 0.888 |
| ENSP00000359206 | BTRC | 15 | 0.89 |
| ENSP00000231509 | NR3C1 | 1 | 0.894 |
| ENSP00000262367 | CREBBP | 9 | 0.898 |
| ENSP00000244741 | CDKN1A | 8 | 0.904 |
| ENSP00000344456 | CTNNB1 | 245 | 0.928 |
| ENSP00000417281 | MDM2 | 97 | 0.932 |
| ENSP00000269571 | ERBB2 | 64 | 0.956 |
| ENSP00000344818 | UBC | 240 | 0.984 |
| ENSP00000275493 | EGFR | 162 | 0.998 |
